# Machine Learning–Based Sleep Electroencephalographic Brain Age Index and Dementia Risk: An Individual Participant Data Meta-Analysis

**DOI:** 10.1001/jamanetworkopen.2026.1521

**Published:** 2026-03-19

**Authors:** Haoqi Sun, Sasha Milton, Yi Fang, Hash Brown Taha, Shreya Shiju, Robert J. Thomas, Wolfgang Ganglberger, Matthew P. Pase, Timothy Hughes, Shaun Purcell, Susan Redline, Katie L. Stone, Kristine Yaffe, M. Brandon Westover, Yue Leng

**Affiliations:** 1 Department of Neurology, Beth Israel Deaconess Medical Center, Boston, Massachusetts; 2 Department of Psychiatry and Behavioral Sciences, University of California, San Francisco; 3 Washington University School of Medicine in St Louis, St Louis, Missouri; 4 Division of Pulmonary, Critical Care, and Sleep Medicine, Department of Medicine, Beth Israel Deaconess Medical Center, Boston, Massachusetts; 5 School of Psychological Science, Monash University, Melbourne, Victoria, Australia; 6 Harvard T. H. Chan School of Public Health, Boston, Massachusetts; 7 Turner Institute for Brain and Mental Health, Monash University, Melbourne, Victoria, Australia; 8 Wake Forest University School of Medicine, Winston-Salem, North Carolina; 9 Department of Psychiatry, Brigham and Women’s Hospital, Boston, Massachusetts; 10 Department of Medicine, Brigham and Women’s Hospital, Boston, Massachusetts; 11 Research Institute, California Pacific Medical Center, San Francisco; 12 Department of Epidemiology, University of California, San Francisco; 13 Department of Neurology, University of California, San Francisco; 14 San Francisco Veterans Affairs Medical Center, San Francisco, California

## Abstract

**Importance:**

Microstructures of sleep electroencephalography (EEG) are closely related to cognition and undergo age-dependent changes. However, their multidimensional nature makes them challenging to interpret using conventional approaches. The machine learning–based EEG brain age index (BAI) measures the deviation between sleep EEG-based brain age and chronological age.

**Objective:**

To determine the association between sleep BAI and incident dementia in community-dwelling populations.

**Data Sources:**

For this individual participant data (IPD) meta-analysis, sleep study data from 5 community-based longitudinal cohorts were pooled. These cohorts included the Multi-Ethnic Study of Atherosclerosis (MESA; 2010-2013), the Atherosclerosis Risk in Communities (ARIC) study (1987-1989), the Framingham Heart Study–Offspring Study (FHS-OS; 1995-1998), the Osteoporotic Fractures in Men Study (MrOS; 2003-2005), and the Study of Osteoporotic Fractures (SOF; 2002-2004).

**Study Selection:**

Adults (aged ≥18 years) without dementia at the time of polysomnography were included.

**Data Extraction and Synthesis:**

The BAI was computed using interpretable machine learning, incorporating sleep EEG features extracted from central channels in overnight, home-based polysomnography. Fine-Gray models were used to assess the association between BAI and incident dementia within each cohort, accounting for death as a competing risk. Cohort-specific estimates were then pooled using random-effects meta-analysis. Analyses were performed between March 2024 and September 2025.

**Main Outcomes and Measures:**

Incident dementia or probable dementia was determined in each cohort, with death as a competing risk.

**Results:**

This meta-analysis included 7105 participants from the MESA (n = 1802; mean [SD] age, 69.3 [9.0] years; 956 females [53.1%]), ARIC (n = 1796; 62.5 [5.7] years; 918 females [51.1%]), FHS-OS (n = 617; 59.5 [8.9] years; 318 females [51.5%]), MrOS (n = 2639 males [100%]; 76.0 [5.3] years), and SOF (n = 251 females [100%]; 82.7 [2.9] years) cohorts. The median (IQR) time to dementia was 4.8 (4.2-5.6) years in the MESA cohort (n = 119 [6.6%]), 16.9 (14.9-19.8) years in the ARIC cohort (n = 354 [19.7%]), 13.1 (8.5-16.2) years in the FHS-OS cohort (n = 59 [9.6%]), 3.6 (1.3-7.1) years in the MrOS cohort (n = 470 [17.8%]), and 4.6 (4.2-5.2) years in the SOF cohort (n = 86 [34.3%]). Across the cohorts, each 10-year increase in BAI was associated with a 39% higher risk of incident dementia (hazard ratio [HR], 1.39 [95% CI, 1.21-1.59]; *P* < .001) after adjustment for covariates. These associations remained after additional adjustment for comorbidities and apnea-hypopnea index scores (HR, 1.31 [95% CI, 1.14-1.50]; *P* < .001) and apolipoprotein E ε4 (HR, 1.22 [95% CI, 1.02-1.45]; *P* = .03), and they were consistent across sex and age groups.

**Conclusions and Relevance:**

In this IPD meta-analysis, a higher sleep EEG-based BAI was associated with a higher risk of incident dementia. These findings highlight the need to evaluate the predictive value of the BAI as a noninvasive digital marker for early detection of dementia in community settings.

## Key Points

**Question:**

Is a higher brain age index (BAI) derived from sleep electroencephalography (EEG) using machine learning associated with a higher risk of future dementia in community-dwelling older adults?

**Findings:**

In this individual participant data meta-analysis of 7105 adults from 5 longitudinal cohorts, every 10-year increase in BAI was associated with a 39% higher risk of incident dementia, independent of age, sex, apolipoprotein E ε4 status, and global cognition and comorbidities at the sleep study.

**Meaning:**

These findings suggest that sleep EEG-based BAI may serve as a promising early digital marker for dementia risk stratification.

## Teaser

This individual participant data meta-analysis explores the association between a machine learning–based sleep electroencephalography (EEG) brain age index and dementia risk among community-dwelling adults from 5 longitudinal cohorts.

## Introduction

Sleep disturbances are increasingly recognized as early indicators and potential modifiable risk factors for dementia.^1,2,3,4,5,6,7^ However, the macrolevel sleep architecture has shown inconsistent associations with cognitive impairment and incident dementia.^8,9^ These broad sleep metrics do not fully capture the complex and multidimensional nature of sleep physiology. In contrast, the microstructure of sleep electroencephalography (EEG) directly reflects the neural processes with explicit functional implications,^7^ presenting an opportunity to develop digital prodromal markers for the early detection of dementia and provide more nuanced insights into cognitive aging.^10,11,12^

Prior studies have shown that cognitive impairment is associated with multiple sleep EEG patterns, including spectral power,^13,14,15^ sleep depth,^16,17^ and spindle–slow oscillation (SO) coupling.^12,13,18^ Despite the promising insights provided by sleep EEG, the vast amount of EEG patterns makes it challenging to summarize and interpret. One innovative approach is to quantify deviations from normal aging patterns. For example, the frequency of the α posterior dominant rhythm peaks at 10 to 11 Hz at around age 30 and gradually decreases to 8 to 9 Hz by age 80,^19^ and spindle density decreases with age.^11,20^

To capture these complex patterns, we developed a sleep EEG-based brain age using a novel, interpretable machine learning approach that integrates multiple age-dependent EEG microstructures into a single agelike number.^21^ The difference between brain age and chronological age is termed the *brain age index* (BAI). An older sleep EEG-based BAI was associated with dementia in a previous clinical-based cross-sectional study.^22^ However, it remains unclear whether BAI is associated with incident dementia in community-dwelling populations.

Here, we computed the BAI from sleep EEG microstructures^21^ and examined its association with incident dementia across 5 community-dwelling longitudinal cohorts using individual participant data (IPD) and random-effects meta-analysis. We examined whether the association between BAI and dementia risk differed by age and sex, and whether key dementia risk factors influenced this association. Additionally, we examined the association between individual EEG features of BAI and dementia risk to interpret their relative contributions.

## Methods

### Study Design

We performed an IPD meta-analysis of 5 prospective cohorts, including the Multi-Ethnic Study of Atherosclerosis (MESA; 2010-2013),^23^ the Atherosclerosis Risk in Communities (ARIC) study (1987-1989),^24^ the Framingham Heart Study–Offspring Study (FHS-OS; 1995-1998) (ie, FHS Gen 2),^25^ the Osteoporotic Fractures in Men Study (MrOS; 2003-2005),^26,27^ and the Study of Osteoporotic Fractures (SOF; 2002-2004).^28^ Details of each cohort are provided in the eMethods in Supplement 1. All cohort committees approved the use of the data. Written informed consent was obtained in each cohort. The MESA study is overseen by a single institutional review board (IRB) at the University of Washington, the ARIC study is overseen at Johns Hopkins University, the FHS-OS is overseen by the Boston Medical Center and Boston University Medical Campus IRB, and the MrOS and SOF studies are overseen at each clinical center or site. These analyses followed the Strengthening the Reporting of Observational Studies in Epidemiology (STROBE) and Meta-Analyses of Observational Studies in Epidemiology (MOOSE) reporting guidelines.

**Figure 1.**
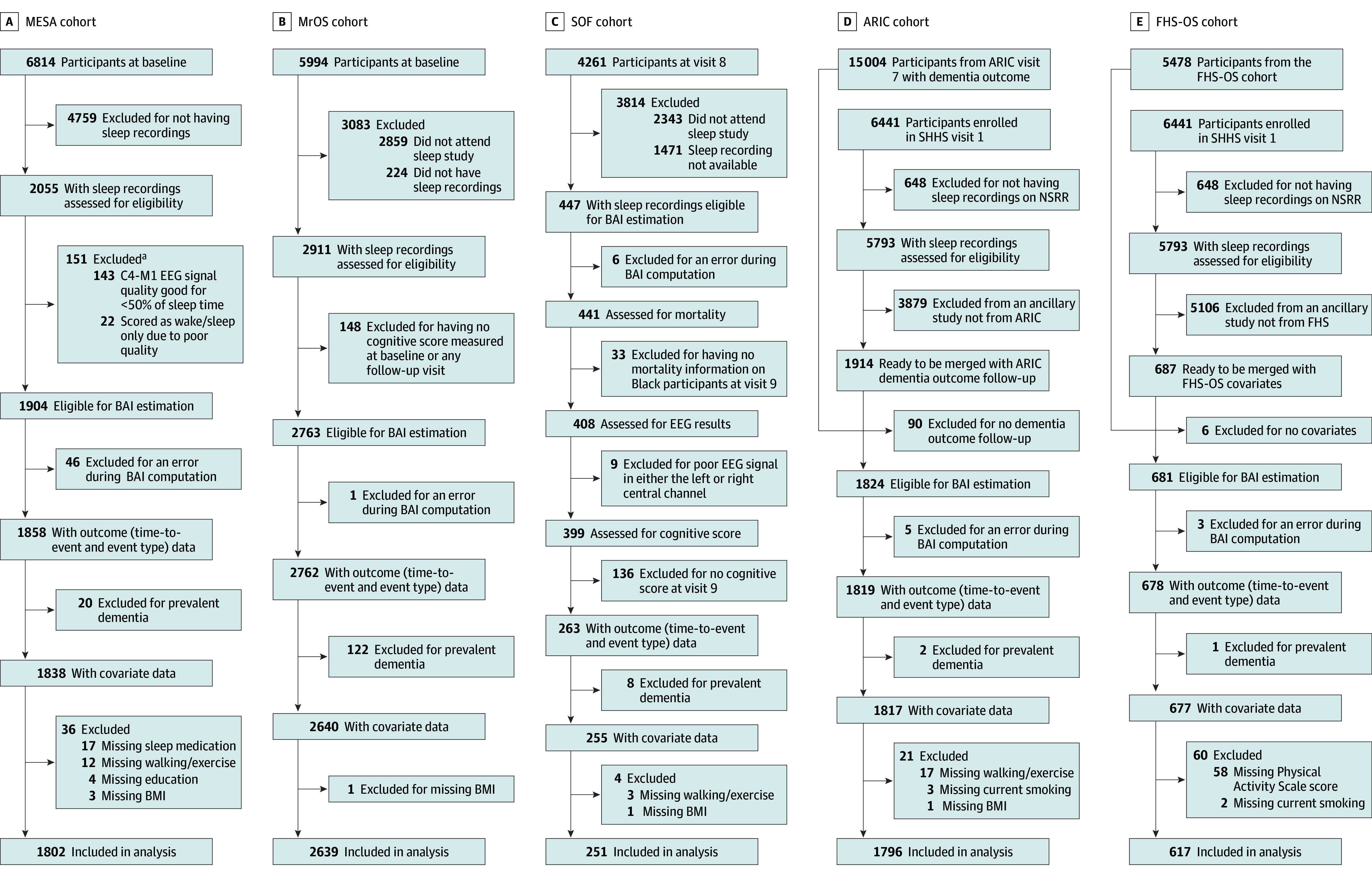
Flowchart of Included Studies ARIC indicates Atherosclerosis Risk in Communities; BAI, brain age index; BMI, body mass index; EEG, electroencephalography; FHS-OS, Framingham Heart Study–Offspring Study; MESA, Multi-Ethnic Study of Atherosclerosis; MrOS, Osteoporotic Fractures in Men; NSRR, National Sleep Research Resource; SHHS, Sleep Heart Health Study; SOF, Study of Osteoporotic Fractures. ^a^The subsample numbers are not mutually exclusive.

As illustrated in Figure 1, the inclusion criteria were (1) availability of overnight sleep polysomnography (PSG) data and (2) availability of outcome data, including time-to-event data and event type (dementia, death, or censored). The exclusion criteria were (1) missing BAI due to the absence of spindles or the presence of excessive artifacts (defined as epochs with amplitude >500 µV or an SD <0.1 µV), (2) missing covariates, and (3) prevalent dementia at the time of the sleep study. Researchers in each cohort recorded participants’ self-reported race and ethnicity as Asian, Black, Hispanic, White, or other race or ethnicity (which includes American Indian or Alaska Native, Native Hawaiian or Other Pacific Islander, multiple races or ethnicities, or unknown race or ethnicity). These data were obtained because race and ethnicity was a relevant biological factor in each cohort.

### Outcomes

The primary outcome was incident dementia, with death treated as a competing risk. Details on both dementia and death ascertainment are provided in the eMethods in Supplement 1. The methods used to determine dementia varied across cohorts and included both clinical adjudication and cognitive score-based approaches at periodic follow-ups. Briefly, in the ARIC, FHS-OS, and SOF studies, dementia adjudication was made by expert panels using serial neuropsychological tests and informant interviews, involving *Diagnostic and Statistical Manual of Mental Disorders, Fifth Edition*^29^ and other criteria as well as a decrease in Mini-Mental State Examination (MMSE) score. In the MESA study, dementia was based on hospitalized *International Classification of Diseases, Ninth Revision* (*ICD-9*) and* Tenth Revision* (*ICD-10*) codes for all-cause dementia^30^ or a decrease in Cognitive Abilities Screening Instrument (CASI) score (a global cognitive score)^31^ of 1.5 SDs or more across participants from examination 5 (near the sleep study) to examination 6. In the MrOS study, probable dementia was ascertained based on self-report of physician diagnosis, dementia medication use, or a decrease in Modified Mini-Mental State Examination (3MS)^32^ score of 1.5 SDs or more across participants from first enrollment to any follow-up visit.

### Sleep EEG-Based BAI

Participants underwent unattended in-home overnight PSG. We used the same EEG preprocessing and brain age computation as described previously^21^ (eMethods in Supplement 1). Briefly, brain age was based on an alternative version of Sun et al^21^ as implemented in Luna, version 0.99.^11^ The features (illustrated in eFigure 1 in Supplement 1) were extracted from artifact-free 30-second epochs, including the following: spindle density; spindle-SO coupling overlap; α (8-12 Hz) band power in N1; δ (0.5-4 Hz), θ (4-8 Hz), α (8-12 Hz), and σ (11-15 Hz) band power kurtosis in N2; δ band power in N3; θ band power kurtosis in N3; δ-to-α ratio and δ-to-θ ratio in N3; and signal waveform kurtosis in N2 and N3. These features were input into a trained brain age model, which outputs brain age as a weighted sum of the EEG microstructures. The model was trained on brain-healthy participants aged 18 to 80 years from a clinical cohort without major neurological or psychiatric diseases, including neurodegenerative diseases, stroke, and epilepsy.^11^ The BAI was defined as brain age minus age, with negative values indicating a younger brain age and positive values indicating an older brain age.

### Statistical Analysis

Within each cohort, we used the Fine-Gray subdistribution hazard model^33,34^ to assess the association between BAI and incident dementia, treating death as a competing risk. Covariates included age at the time of the sleep study, sex, race and ethnicity, education, body mass index (BMI), current smoking, sleep medication use, and physical activity level. eTable 1 in Supplement 1 provides definitions of these covariates for each cohort, noting that some covariates were excluded in certain cohorts and that others were defined differently across cohorts. We fit 2 models for each cohort: (1) a minimally adjusted model (adjusted for age and sex) and (2) a fully adjusted model (additionally adjusted for race and ethnicity, education, BMI, current smoking, sleep medication use, and physical activity level). For each cohort, we estimated the hazard ratios (HRs) and 95% CIs for a 10-year increase in BAI. We applied Fine-Gray models to examine associations between individual BAI features and dementia risk, with HRs calculated per 1-SD increase in each feature.

In sensitivity analyses, we adjusted for the following additional covariates where available: global cognition scores at the time of the sleep assessment; a diagnosis of depression, diabetes, hypertension, myocardial infarction, or stroke at the sleep study; apnea-hypopnea index (AHI) score (3% rule for hypopnea); and apolipoprotein E (*APOE*) ε4 allele carrier status. Global cognition scores at the sleep study were assessed using the CASI in the MESA cohort (≥77 was the median cutoff for cognitively normal across all sites in Teng et al^31^), the 3MS in the MrOS cohort (≥81 for cognitively normal^32^), and the MMSE in the ARIC, FHS-OS, and SOF cohorts (≥24 for cognitively normal). We performed stratified analyses in males and females and in younger (<70 years) and older (≥70 years) individuals (because 70 years was the overall mean age). Cohorts without eligible participants for a given subgroup were excluded from the corresponding analyses.

Cohort-specific estimates were pooled using an inverse-variance random-effects model following the Der Simonian and Laird model.^35^ The Higgins *I*^2^ test was used to assess heterogeneity across cohorts.^36^ The IPD meta-analysis approach mitigated the harmonization problem of the cohort-specific covariates. The analyses were performed using the meta package,^37^ version 7.0.0, in R, version 4.3.2 (R Project for Statistical Computing), which includes the statistical tests for *P* values. Two-sided *P* < .05 was considered significant. Analyses were performed between March 2024 and September 2025.

## Results

### Cohort Characteristics

This study included 7105 participants from the 5 cohorts as follows (Table). The 1802 participants in the MESA cohort^23^ (956 females [53.1%] and 846 males [46.9%]) had a mean (SD) age of 69.3 (9.0) years; 211 (11.7%) were Asian, 499 (27.7%) were Black, 420 (23.3%) were Hispanic, and 672 (37.3%) were White. The 1796 participants in the ARIC cohort^24^ (918 females [51.1%] and 878 males [48.9%]) had a mean (SD) age of 62.5 (5.7) years; all (100%) were White. The 617 participants in the FHS-OS cohort^25^ (318 females [51.5%] and 299 males [48.5%]) had a mean (SD) age of 59.5 (8.9) years; 1 (0.2%) was Hispanic, 554 (89.8%) were White, and 62 (10.0%) were of other race or ethnicity. The MrOS cohort^26,27^ comprised 2639 males (100%), with a mean age of 76.0 (5.3) years; 74 (2.8%) were Asian, 76 (2.9%) were Black, 2422 (91.8%) were White, and 67 (2.5%) were of other race or ethnicity. Finally, the SOF cohort^28^ comprised 251 females (100%), with a mean age of 82.7 (2.9) years; 250 participants (99.6%) were White, and 1 (0.4%) was of other race or ethnicity.

**Table.** Participant Characteristics Across All Cohorts Included in the Meta-Analysis^a^

Characteristic	Cohort (N = 7105)				
	MESA (n = 1802)	ARIC (n = 1796)	FHS-OS (n = 617)	MrOS (n = 2639)	SOF (n = 251)
Age at sleep study, mean (SD), y	69.3 (9.0)	62.5 (5.7)	59.5 (8.9)	76.0 (5.3)	82.7 (2.9)
Sex					
Female	956 (53.1)	918 (51.1)	318 (51.5)	0	251 (100)
Male	846 (46.9)	878 (48.9)	299 (48.5)	2639 (100)	0
Race and ethnicity					
Asian	211 (11.7)	0	0	74 (2.8)	0
Black	499 (27.7)	0	0	76 (2.9)	0
Hispanic	420 (23.3)	NA	1 (0.2)	NA	NA
White	672 (37.3)	1796 (100)	554 (89.8)	2422 (91.8)	250 (99.6)
Other race or ethnicity^b^	0	0	62 (10.0)	67 (2.5)	1 (0.4)
College degree	726 (40.3)	791 (44.0)	386 (62.6)	2106 (79.8)	200 (79.7)
BMI, median (IQR)	27.8 (24.7-31.7)	28.2 (25.2-31.5)	27.8 (25.2-31.3)	26.8 (24.6-29.3)	27.1 (24.6-30.6)
Cognitive score at sleep study					
Assessment used (score cutoff for cognitively normal status)	CASI (≥77)	MMSE (≥24)	MMSE (≥24)	3MS (≥81)	MMSE (≥24)
Score, median (IQR)	90 (83-94)	29 (27-30)	29 (28-30)	94 (91-97)	29 (28-30)
Cognitively normal participants	1620 (89.9)	1704 (94.9)	610 (98.9)	2615 (99.1)	250 (99.6)
Current smoker	125 (6.9)	183 (10.2)	72 (11.7)	55 (2.1)	3 (1.2)
*APOE* ε4 carrier status among available^c^	467/1717 (27.2)	NA	81/386 (21.0)	553/2326 (23.8)	11/139 (7.9)^d^
Incident dementia	119 (6.6)	354 (19.7)	59 (9.6)	470 (17.8)	86 (34.3)
Time to dementia from sleep study, median (IQR), y	4.8 (4.2-5.6)	16.9 (14.9-19.8)	13.1 (8.5-16.2)	3.6 (1.3-7.1)	4.6 (4.2-5.2)
BAI, mean (SD), y	−4.2 (6.1)	−0.0 (6.0)	0.6 (5.8)	−3.4 (5.6)	−5.4 (5.7)
AHI, median (IQR), events/h	8.6 (3.2-19.4)	5.2 (1.9-13.0)	4.5 (1.5-11.7)	8.1 (3.2-16.6)	9.6 (5.2-18.8)
Sleep medication use at sleep study	200 (11.1)	189 (10.5)	50 (8.1)	358 (13.6)	48 (19.1)
Hypertension at sleep study	1015 (56.3)	651 (36.2)	204 (33.1)	1299 (49.2)	148 (59.0)
Diabetes at sleep study	345 (19.1)	97 (5.4)	35 (5.7)	340 (12.9)	26 (10.4)
Myocardial infarction at sleep study	35 (1.9)	112 (6.2)	33 (5.3)	445 (16.9)	33 (13.1)
Stroke at sleep study	25 (1.4)	42 (2.3)	7 (1.1)	99 (3.8)	32 (12.7)
Depression at sleep study	253 (14.0)	40 (2.2)	23 (3.7)	142 (5.4)	23 (9.2)

Abbreviations: 3MS, Modified Mini-Mental State Examination; AHI, Apnea-Hypopnea Index; *APOE*, apolipoprotein E; ARIC, Atherosclerosis Risk in Communities; BAI, Sleep EEG-Based Brain Age Index; BMI, body mass index (calculated as weight in kilograms divided by height in meters squared); CASI, Cognitive Abilities Screening Instrument; EEG, electroencephalography; FHS-OS, Framingham Heart Study–Offspring Study; MESA, Multi-Ethnic Study of Atherosclerosis; MMSE, Mini-Mental State Examination; MrOS, Osteoporotic Fractures in Men; NA, not available; SOF, Study of Osteoporotic Fractures.

^a^ Unless indicated otherwise, values are presented as No. (%) of participants.

^b^ Includes American Indian or Alaska Native, Native Hawaiian or Other Pacific Islander, multiple races or ethnicities, or unknown race or ethnicity.

^c^ Genotype data were available for 1717 participants (95.3%) in the MESA cohort, 0 in the ARIC cohort, 386 (62.6%) in the FHS-OS cohort, 2326 (88.1%) in the MrOS cohort, and 139 (55.4%) in the SOF cohort.

^d^ SOF participants with *APOE *genotyping came from only 1 study site.

At the time of sleep assessment, the majority of participants (MESA: n = 1620 [89.9%]; ARIC: n = 1704 [94.9%]; FHS-OS: n = 610 [98.9%]; MrOS: n = 2615 [99.1%]; and SOF: n = 250 [99.6%]) across cohorts were cognitively normal. The median (IQR) time to dementia was 4.8 (4.2-5.6) years in the MESA cohort (n = 119 [6.6%]), 16.9 (14.9-19.8) years in the ARIC cohort (n = 354 [19.7%]), 13.1 (8.5-16.2) years in the FHS-OS cohort (n = 59 [9.6%]), 3.6 (1.3-7.1) years in the MrOS cohort (n = 470 [17.8%]), and 4.6 (4.2-5.2) years in the SOF cohort (n = 86 [34.3%]). There was a significant difference in dementia incidence rates across cohorts (*P* < .001, χ^2^ test), and differences in demographic composition of the cohorts may partially account for this variation. eTable 2 in Supplement 1 presents the cumulative number of participants who developed dementia or died over 25 years. The mean (SD) BAI was −4.2 (6.1) years in the MESA cohort, −0.0 (6.0) years in the ARIC cohort, 0.6 (5.8) years in the FHS-OS cohort, −3.4 (5.6) years in the MrOS cohort, and −5.4 (5.7) years in the SOF cohort.

As illustrated in Figure 1, a small number of participants were excluded from BAI estimation due to the absence of Luna-detected spindles or the presence of excessive EEG artifacts (MESA: n = 46; ARIC: n = 5; FHS-OS: n = 3; MrOS: n = 1; and SOF: n = 6). In the MESA cohort—the only cohort with more than a few exclusions—participants missing a BAI did not differ in age (mean [SD], 70.6 [10.2] vs 69.5 [9.1] years; *P* = .42) or sex (25 [58.1%] vs 985 [53.0%] female; *P* = .51) compared with those included. Given the minimal exclusions in other cohorts, this level of missingness was unlikely to affect the results or generalizability.

**Figure 2.**
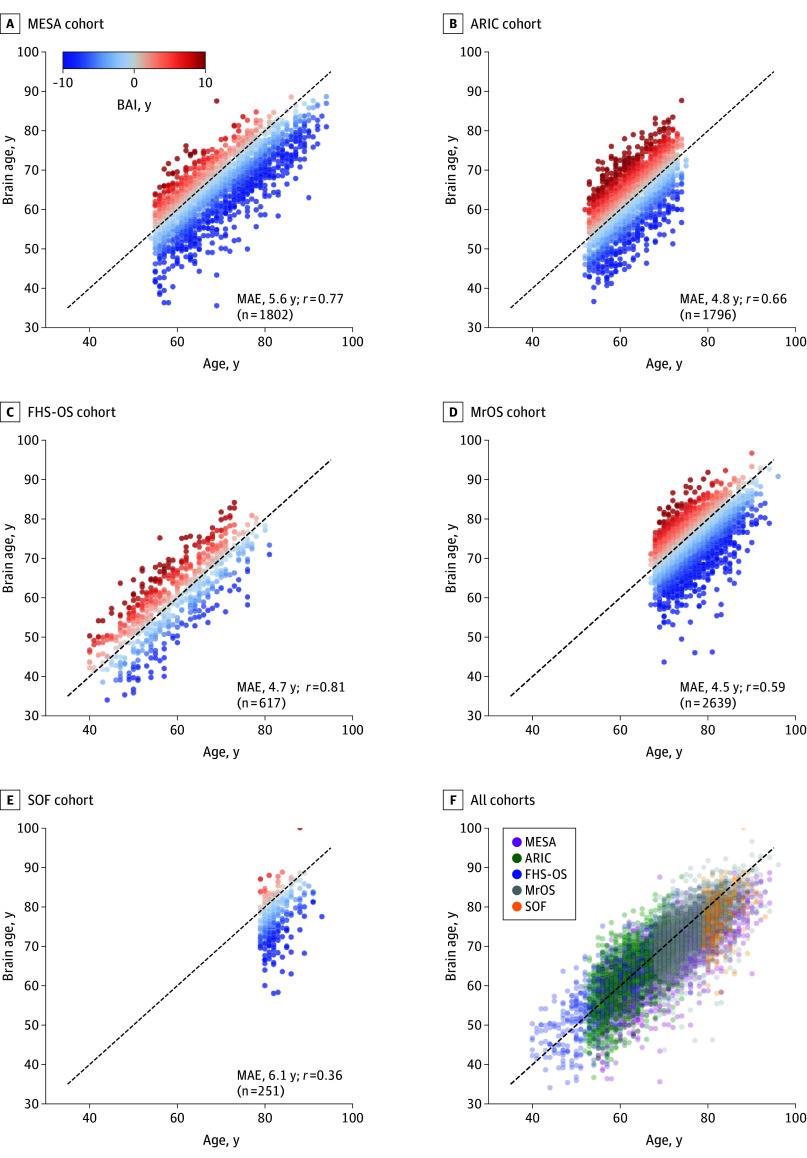
Scatter Plots of Chronological Age vs Sleep Electroencephalography (EEG)–Based Brain Age in All Cohorts Each dot represents 1 participant. The diagonal line represents where age equals brain age. All cohorts are overlaid in panel F. ARIC indicates Atherosclerosis Risk in Communities; FHS-OS, Framingham Heart Study–Offspring Study; MAE, mean absolute error; MESA, Multi-Ethnic Study of Atherosclerosis; MrOS, Osteoporotic Fractures in Men; SOF, Study of Osteoporotic Fractures.

As shown in Figure 2, sleep EEG-based brain ages had mean absolute errors (MAEs) of 5.6 years in the MESA cohort, 4.8 in the ARIC cohort, 4.7 in the FHS-OS cohort, 4.5 in the MrOS cohort, and 6.0 in the SOF cohort. eTable 3 in Supplement 1 presents the characteristics of participants with lower BAI (≤−3 years) and higher BAI (≥3 years) and their univariate comparison results. BAI was negatively associated with global cognitive scores at the sleep study in 2 cohorts, although the correlations were small (ARIC: *r* = −0.06, *P* = .029; and MrOS: *r* = −0.05, *P* = .012). No associations were observed in the MESA, FHS-OS, and SOF cohorts.

### Association Between BAI and Incident Dementia

**Figure 3.**
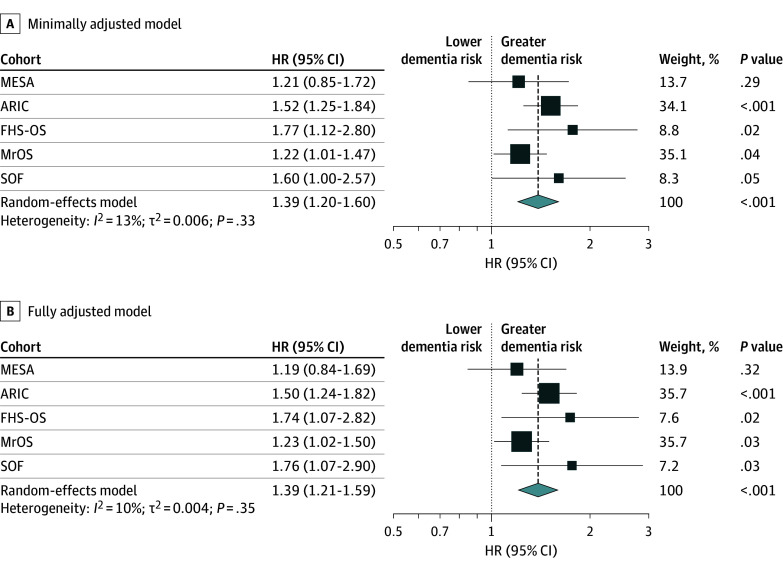
Pooled Hazard Ratios (HRs) for Associations Between Brain Age Index and Incident Dementia A, Adjusted for age and sex. B, Adjusted for age, sex, education, body mass index, current smoking, race, sleep medication use, and physical activity level. ARIC indicates Atherosclerosis Risk in Communities; FHS-OS, Framingham Heart Study–Offspring Study; MESA, Multi-Ethnic Study of Atherosclerosis; MrOS, Osteoporotic Fractures in Men; SOF, Study of Osteoporotic Fractures.

There were 1082 participants with incident dementia in total. For the minimally adjusted model, each 10-year increase in BAI was associated with a 39% higher risk of incident dementia (HR, 1.39 [95% CI, 1.20-1.60]; *P* < .001 [*I*^2^ = 13%; *P* = .33]) (Figure 3A). After adjusting for age, sex, education, BMI, current smoking, race and ethnicity, sleep medication use, and physical activity level, each 10-year increase in BAI was still associated with a 39% higher risk of incident dementia (HR, 1.39 [95% CI, 1.21-1.59]; *P* < .001 [*I*^2^ = 10%; *P* = .35]) (Figure 3B). In the fully adjusted model, HRs across individual cohorts were 1.19 (95% CI, 0.84-1.69) in the MESA cohort, 1.50 (95% CI, 1.24-1.82) in the ARIC cohort, 1.74 (95% CI, 1.07-2.82) in the FHS-OS cohort, 1.23 (95% CI, 1.02-1.50) in the MrOS cohort, and 1.76 (95% CI, 1.07-2.90) in the SOF cohort. Adding polynomials of BAI resulted in a better fit (lower bayesian information criterion) in only 1 cohort (SOF) and no improvement in the other 4 cohorts.

**Figure 4.**
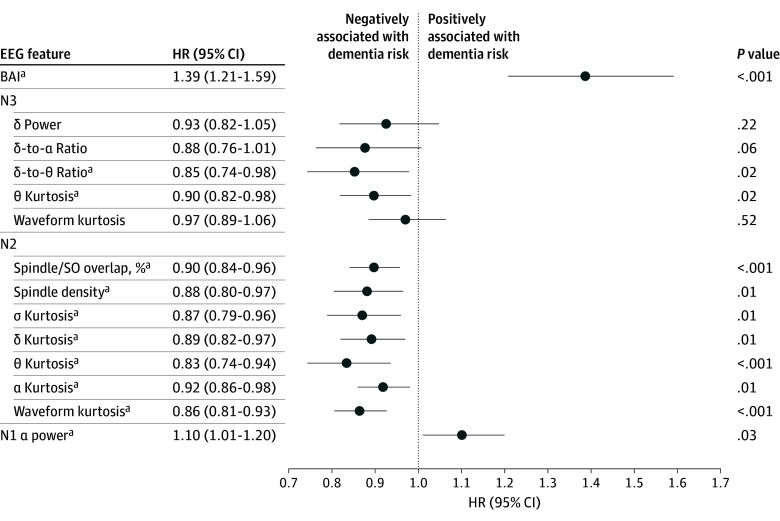
Pooled Hazard Ratios (HRs) for Associations Between Electroencephalography (EEG) Features Contributing to Brain Age Index (BAI) and Incident Dementia HRs greater than 1 indicate a positive association with dementia risk, whereas HRs less than 1 indicate a negative association. BAI is per 10-year increase, whereas BAI features are per 1-SD increase. ^a^Feature associated with incident dementia.

Under the full adjustment model, we examined the individual sleep EEG microstructure features that contribute to BAI (Figure 4). Of the 13 features studied, 10 showed associations with incident dementia in the pooled analyses, including ɑ power in N1; spindle-SO overlap, spindle density, σ kurtosis, δ kurtosis, θ kurtosis, ɑ kurtosis, and waveform kurtosis in N2; and δ-to-θ ratio and θ kurtosis in N3. Features from N2 and N3 were negatively associated with incident dementia, whereas the only N1 feature was positively associated with incident dementia. The top feature in terms of statistical significance was waveform kurtosis in N2 (HR, 0.86 [95% CI, 0.81-0.93] for 1 SD; *P* < .001), which was negatively associated with incident dementia and likely reflected K-complex activity—that is, large, high-amplitude events that produce heavy-tailed amplitude distributions and are characterized by high kurtosis.

### Association Between BAI and Dementia in Sensitivity Analyses

After further adjustment for cognitive score, diabetes, hypertension, myocardial infarction, stroke, depression, and AHI score at the time of the sleep assessment, the association between BAI and incident dementia was slightly attenuated but remained (HR, 1.31 [95% CI, 1.14-1.50]; *P* < .001) (eFigure 2 in Supplement 1). These results suggest that the association of BAI with subsequent dementia is independent of cognitive status and several comorbidities at the sleep study. Regarding the *APOE* ε4 allele, the association remained after adjusting for *APOE* ε4 carrier status (HR, 1.22 [95% CI, 1.02-1.45]; *P* = .03) compared with the HR (1.23 [95% CI, 1.04-1.46]; *P* = .02) in the same subset without this adjustment (eFigure 3 in Supplement 1).

There was no interaction between age and BAI (HR, 1.06 [95% CI, 0.55-2.06]; *P* = .86), between sex and BAI (HR, 0.88 [95% CI, 0.49-1.57]; *P* = .66), or between *APOE* ε4 carrier status and BAI (HR, 0.94 [95% CI, 0.69-1.28]; *P* = .70). Stratified analyses showed that the HR was 1.65 in females (95% CI, 1.13-2.40; *P* = .01 [*I*^2^ = 66%; *P* = .03]; n = 2447 without and 380 with incident dementia) (eFigure 4A in Supplement 1) and 1.25 in males (95% CI, 1.07-1.46; *P* = .005 [*I*^2^ = 0; *P* = .89]; n = 4624 without and 702 with incident dementia) (eFigure 4B in Supplement 1). Heterogeneity was observed only in the female-only analysis. After the MESA cohort was excluded, heterogeneity was no longer present, and the HR increased to 1.86 (95% CI, 1.52-2.28; *P* < .001). The associations were similar among individuals younger than 70 years (HR, 1.43 [95% CI, 1.11-1.83]; *P* = .006 [*I*^2^ = 16%; *P* = .31]; n = 3288 without and 338 with incident dementia) (eFigure 4C in Supplement 1) and those aged 70 years or older (HR, 1.34 [1.12-1.60]; *P* = .001 [*I*^2^ = 41%; *P* = .15]; n = 3783 without and 744 with incident dementia) (eFigure 4D in Supplement 1).

Since the dementia outcome in the MESA cohort was ascertained using hospitalization *ICD-9* and *ICD-10* codes, which have limited sensitivity and may introduce bias, we also performed sensitivity analyses excluding MESA. As shown in eFigure 5A in Supplement 1, the association remained, with an HR of 1.43 (95% CI, 1.21-1.69; *P* < .001 [*I*^2^ = 19%; *P* = .30]). Furthermore, because death ascertainment in the MESA and ARIC cohorts was primarily based on hospital records, we conducted additional analyses excluding these cohorts. The association remained (eFigure 5B in Supplement 1), with an HR of 1.44 (95% CI, 1.10-1.88; *P* < .01 [*I*^2^ = 32%; *P* = .23]).

## Discussion

In this meta-analysis of 7105 participants across 5 community-based cohorts,^23,24,25,26,27,28^ higher sleep EEG-based BAI was consistently associated with a higher risk of incident dementia over a median follow-up ranging from 3.6 to 16.9 years. Each 10-year increase in BAI was associated with a 39% higher risk of incident dementia. This association was independent of baseline cognitive status and remained robust in various sensitivity analyses and stratified analyses. While our prior work has validated sleep-based BAI in a clinical setting,^22^ the present findings show—for the first time, to our knowledge—that its association with future dementia risk extends to community-dwelling populations.

The key strengths of this study include the large, pooled sample comprising diverse longitudinal cohorts with harmonized sleep protocols; an interpretable machine learning approach that integrates rich, multidimensional sleep EEG features into a simple marker; and a random-effects meta-analysis to address between-study heterogeneity. Here, we applied a brain age framework to evaluate BAI as a generalizable and biologically interpretable marker of neurophysiological aging. Unlike a direct dementia prediction model, BAI is trained on large lifespan EEG datasets using chronological age as the target, allowing us to leverage substantially larger and more diverse EEG data than would be available for dementia outcomes and to derive a stable, transferable representation of normative brain aging. Therefore, the observed association between higher BAI and incident dementia provides an important validation of BAI as a marker of accelerated brain aging.

The association between incident dementia and BAI based on sleep EEG microstructures (features) provides more granular information than the conventional sleep macrostructure measures. A prior pooled analysis,^9^ including 4 of the 5 cohorts used in our study, found no associations between incident dementia and a traditional summary of sleep measures such as time spent in different sleep stages (pooled HRs for percentages for N1, N2, N3, and rapid eye movement [REM] ranged from 0.98 to 1.03; all *P* > .05), wake after sleep onset, sleep efficiency, AHI, or relative δ power. These results suggested that traditional sleep macrostructure measures may not adequately capture the neurophysiological changes relevant to cognitive decline. This null result may reflect the somewhat arbitrary nature of several standard sleep measures, including 30-second scoring epochs and the criteria for slow waves to differentiate N2 and N3. Our study extends prior cross-sectional work in the MESA and MrOS studies that compared sleep macrostructure and EEG microstructure with cognitive performance.^12^ While that study found EEG microstructures to be more associated with cognition than macrostructure measures such as REM duration and sleep efficiency, the large number of EEG microstructure features limited clinical applicability. In contrast, we derived a single machine learning–based BAI that summarizes microstructural information and is associated with future dementia risk, advancing from cross-sectional associations to a longitudinal, potentially prognostic marker.

Several neurobiological pathways may underlie the association between elevated BAI and higher dementia risk. Structural MRI–EEG studies show that degeneration of the thalamus and hippocampus—regions essential for spindle generation and memory consolidation—is associated with reductions in σ power and spindle activity.^10^ Higher cerebrospinal fluid tau and greater amyloid burden have also been associated with alterations in spindle activity, slow oscillations, and EEG slowing.^38^ Moreover, reduced perfusion in frontal, cingulate, and precuneus regions has been associated with REM microstructure, including decreased δ power and increased α and β power.^39^ In our study, adjustment for *APOE* genotype produced only minimal changes in the association between BAI and incident dementia, indicating that BAI is not simply a proxy for Alzheimer disease (AD) genetic susceptibility. However, given the observational nature of the study, we cannot determine whether sleep EEG alterations contribute directly to AD pathology or whether individuals with elevated BAI already harbor subclinical AD-related changes.

### Limitations

This study has several limitations. First, the 5 cohorts included in this study differ in population characteristics, data collection methodologies, dementia ascertainment procedures, and follow-up durations, which may introduce heterogeneity and potential bias when pooling results across studies. For instance, in the MESA study,^23^ 86% of dementia outcomes were identified using *ICD-9* and *ICD-10* codes and death certificates from hospitalization, which may underreport true dementia cases; the remaining 14% were based on large declines in CASI scores (>1.5-SD decline) over 6 years, which does not reflect the actual time of dementia onset. In the SOF study,^28^ cognitive status was adjudicated only once, approximately 4 to 5 years after PSG, where the time to diagnosis had to be approximated by time to adjudication, limiting precision in dementia onset time. While such differences may affect comparability, we addressed this issue through random-effects meta-analysis, heterogeneity testing, and subgroup analyses. Moreover, sensitivity analyses excluding cohorts with unique dementia or death ascertainment methods (eg, MESA and ARIC) yielded consistent results, underscoring the robustness of the observed association.

Second, only death was used as a competing risk for dementia. However, other life events—such as major surgeries, psychiatric illnesses, or acute medical conditions—may also affect follow-up or dementia ascertainment and warrant consideration as additional competing risks.

Third, due to the observational nature of these studies, we cannot infer a causal relationship between BAI and dementia. Moreover, as a composite measure, BAI itself is not a plausible therapeutic target. Rather, BAI should be viewed as a prognostic marker for future dementia risk. When an individual’s BAI is older than expected, indicating elevated risk, the next step is to examine the specific EEG microstructural components driving this deviation, interpret their neurophysiological significance, and assess whether these underlying processes may represent viable therapeutic targets. A related limitation is our reliance on EEG microstructural features for BAI estimation. Although these features offer more granular information than macrostructural sleep measures, they may limit broader applicability. For future work, validation using wearable devices is needed to ensure broader generalizability and support clinical implementation, and further investigation is warranted to determine how sleep microstructures interact with macrostructural measures.

## Conclusions

In this IPD meta-analysis, we found that an elevated sleep EEG-based BAI, a machine learning marker of brain aging, was independently associated with higher dementia risk in community-based populations. Using interpretable EEG microstructures, BAI offered insights into neurophysiological signals that reflected future dementia risk or resilience. Beyond risk prediction, BAI may help identify individuals who warrant closer cognitive monitoring and could enrich higher-risk populations for prevention trials. Longitudinal tracking of BAI trajectories may further inform when additional diagnostic evaluation is appropriate. With future validation in wearable EEG devices, BAI has the potential to complement emerging plasma and imaging markers, supporting multimodal risk stratification to better inform clinical decision-making.

## Notes

Supplement 1.
eMethods

eReferences
**eTable 1.** Covariates Used in Each Cohort **eTable 2.** Cumulative Number of Participants Who Developed Dementia or Died Over 25 Years **eTable 3.** Participant Characteristics for Low Brain Age Index (BAI) (≤ –3 Years) vs High BAI (≥3 Years) **eFigure 1.** Illustration of Example Sleep Electroencephalographic Microstructure Features **eFigure 2.** Forest Plots of Associations Between Brain Age Index and Incident Dementia While Adjusting for Many (Overcomplete) Covariates **eFigure 3.** Forest Plots of Associations Between Brain Age Index and Dementia While Adjusting and Not Adjusting for *APOE* ε4 Allele Carrier Status **eFigure 4.** Forest Plots of Associations Between Brain Age Index and Incident Dementia Stratified by Sex and Age **eFigure 5.** Forest Plots of the Associations Between Brain Age Index and Dementia While Excluding the MESA Cohort

Supplement 2.
Data Sharing Statement
