# Supplementary material for: Machine Learning–Based Sleep Electroencephalographic Brain Age Index and Dementia Risk: An Individual Participant Data Meta-Analysis
Source: JAMA Netw Open. 2026 Mar 19;9(3):e261521. doi: 10.1001/jamanetworkopen.2026.1521 (PMC13003368; doi:10.1001/jamanetworkopen.2026.1521)
Supplement: Supplement 1. — eMethods eReferences eTable 1. Covariates Used in Each Cohort eTable 2. Cumulative Number of Participants Who Developed Dementia or Died Over 25 Years eTable 3. Participant Characteristics for Low Brain Age Index (BAI) (≤ –3 Years) vs High BAI (≥3 Years) eFigure 1. Illustration of Example Sleep Electroencephalographic Microstructure Features eFigure 2. Forest Plots of Associations Between Brain Age Index and Incident Dementia While Adjusting for Many (Overcomplete) Covariates eFigure 3. Forest Plots of Associations Between Brain Age Index and Dementia While Adjusting and Not Adjusting for APOE ε4 Allele Carrier Status eFigure 4. Forest Plots of Associations Between Brain Age Index and Incident Dementia Stratified by Sex and Age eFigure 5. Forest Plots of the Associations Between Brain Age Index and Dementia While Excluding the MESA Cohort [file jamanetwopen-e261521-s001.pdf]

Supplementary Online Content

Sun H, Milton S, Fang Y, et al. Machine learning–based sleep electroencephalographic brain age index and dementia risk: an individual participant data meta-analysis. *JAMA Netw Open*. 2026;9(3):e261521. doi:10.1001/jamanetworkopen.2026.1521

eMethods

eReferences

eTable 1. Covariates Used in Each Cohort

eTable 2. Cumulative Number of Participants Who Developed Dementia or Died Over 25 Years

eTable 3. Participant Characteristics for Low Brain Age Index (BAI) (≤ –3 Years) vs High BAI (≥3 Years)

eFigure 1. Illustration of Example Sleep Electroencephalographic Microstructure Features

eFigure 2. Forest Plots of Associations Between Brain Age Index and Incident Dementia While Adjusting for Many (Overcomplete) Covariates

eFigure 3. Forest Plots of Associations Between Brain Age Index and Dementia While Adjusting and Not Adjusting for *APOE* ε4 Allele Carrier Status

eFigure 4. Forest Plots of Associations Between Brain Age Index and Incident Dementia Stratified by Sex and Age

eFigure 5. Forest Plots of the Associations Between Brain Age Index and Dementia While Excluding the MESA Cohort

This supplementary material has been provided by the authors to give readers additional information about their work.

© 2026 Sun H et al. *JAMA Network Open*.

eMethods

Study Design

For all cohorts, sleep data were collected using a similar protocol overseen by the same sleep reading center, where data were processed and scored similarly. The sleep studies of MESA were done at 2010-2013 with a median of 4.8 years to dementia, ARIC at 1987-1989 and 16.9 years, FHS-OS at 1995-1998 and 13.1 years, MrOS at 2003-2005 and 3.6 years, and SOF at 2002-2004 and 4.6 years.

MESA is a multi-center, prospective cohort study of Black, White, Hispanic, and Chinese American adults. Between 2010 and 2012, participants were also enrolled in a Sleep Exam (in 2010-2013 in conjunction with MESA Exam 5), which included one full overnight unattended sleep polysomnography (PSG).

ARIC is a multi-center, prospective observational cohort study initially focused on cardiovascular risk factors, medical care, and disease in four US communities, between 1987 and 1989.

The FHS-OS cohort is a community-based prospective observational cohort study that enrolled offspring of the original FHS cohort from 1971 to 1975 in Framingham, Massachusetts[1]. Between 1995 and 1998, participants from the ARIC and FHS-OS cohorts completed overnight home-based polysomnography as part of the Sleep Heart Health Study (SHHS)[2].

MrOS is a prospective study of community-dwelling individuals enrolled from 2000 to 2002 at six clinical centers in the United States. Sleep PSG data were collected between 2003 and 2005.

SOF is a multi-center prospective cohort study of women with sleep studies completed between 2002 and 2004.

The analyses were conducted between March 2024 and September 2025.

Dementia Outcome Ascertainment

The primary outcome for our pooled analysis was incident dementia, with death treated as a competing risk. If neither dementia nor death occurred, the participant was censored at the last contact. The time-to-event was defined as the number of days from the sleep study to the first ascertainment of the event, including dementia, death, or other censoring events. Detailed definitions can be found in each cohort and are briefly described below.

In MESA[3,4], the participants were contacted by telephone interviews (participant or proxy) every 9 to 12 months, and were followed up until 2018. Dementia was assessed using two criteria sequentially: (1) Possible International Classification of Diseases (ICD)-based all-cause dementia as adjudicated by the MESA Cognitive Working Group based on Fujiyoshi et al[5]; and (2) decrease in Cognitive Abilities Screening Instrument (CASI, a global cognitive score)[6] from Exam 5 (close to sleep study) to Exam 6 is $\geq$ 1.5 standard deviations of the changes across participants, which was 9.8 points. The time to dementia is from the sleep study to the first date of hospitalization, meeting any of the ICD-based criteria in case (1); and to Exam 6 in case (2). There is likely a substantial group of false negatives (i.e., outcome is falsely not dementia), from people who were never hospitalized or not known to be dead.

In ARIC[7], the participants were followed up until 2019 (end of Visit 7). Dementia[8] was based on reviewer diagnosis, algorithmic syndromic diagnosis, AD8 Dementia Screening Interview, Six Item Screener, dementia codes on the cohort eligibility form, and dementia codes on the death certificate form (ordered by priority, designated as level 3 in ARIC), while excluding discharge or death certificate codes that occurred after the date of last visit/phone contact. The time to dementia was based on the earliest date of the event and then subtracting 180 days to approximate the midpoint of the prior year when cognitive decline likely began.

In FHS-OS[9], the participants were followed up until 2018. Dementia[10] was based on serial Mini-Mental State Examination (MMSE) from 1991-1998, and neuropsychological tests every 5 or 6 years since 1999. Participants who were identified as having possible dementia based on these screening assessments were invited to undergo additional, annual neurologic and neuropsychological examinations. Additional examinations were performed if the participant or a family member reported subjective cognitive decline. A dementia review panel reviewed every case of possible cognitive decline and dementia ever documented in the FHS, including the date of onset. The diagnosis of dementia was based on criteria from the Diagnostic and Statistical Manual of Mental Disorders (DSM-IV).

In MrOS[11], the participants were followed up until 2016 (end of Visit 4). Dementia was based on the report of physician-diagnosed dementia, self-reported dementia, dementia medication use, or having a change in the Modified Mini-Mental State (3MS) scores[12] ≥ 1.5 standard deviations worse than the mean change from the sleep visit to any follow-up visit (a decline of 7.32, 9.43, and 13.62 points on 3MS from baseline to visit 2, visit 3 and visit 4, respectively). The 3MS is a global measurement of cognitive function, with components for orientation, concentration, language, praxis, and immediate and delayed memory.

In SOF[13], the dementia status was ascertained at Visit 9 (2007-2008). Dementia was identified through a two-stage protocol. First, all participants were screened for the following criteria: (1) score <88 on the 3MS; (2) score <4 on the CVLT delayed recall; (3) score ≥3.6 on the IQCODE; (4) previous dementia diagnosis; or (5) nursing home residence. These participants, along with a small random sample of screen-negative participants, underwent detailed adjudication by a rotating panel of dementia specialists who reviewed neuropsychological test results, functional measures, medication use, longitudinal cognitive data, mood scores, and medical history. The agreement among adjudicators achieved Cohen's κ of 0.77. A diagnosis of dementia was made based on DSM-IV criteria, where the functional loss was based on IQCODE from the informant or, if unavailable, self-reported ADL/IADL limitations.

Death Ascertainment

In MESA[3,4], each participant was contacted by telephone interviews (participant or proxy) every 9 to 12 months to obtain information including death. Death information was also collected from death certificates from hospitals, medical records from hospitalizations, and autopsy reports. National Death Index database was also queried.

In ARIC[7], death was ascertained through annual telephone contact with participant proxy, obituaries, hospital records, death certificates, or vital statistics from the National Death Index.

In FHS-OS[9], all deaths were adjudicated by a panel, which reviews medical and nursing records up to the date of death and the death certificate.

In MrOS[11], participants were contacted every 4 months via postcard to determine vital status. Next of kin were contacted in cases of nonresponse. Reported deaths through 2018 were confirmed by a centralized review of death certificates. We used the death status at the end of Visit 4, the last visit at which cognitive status was obtained.

In SOF[13], deaths were ascertained by contacts every 4 months and confirmed with death certificates. Four clinics individually collect SOF mortality, where a State Registered Certificate of Death was also submitted to the Coordinating Center.

Importantly, participants who experienced neither dementia nor death were censored at the date of last contact. Time-to-event was defined as the number of days from the sleep study to the first occurrence of dementia, death, or censoring.

Details on Sleep EEG-Based Brain Age Index

Here is a summary of the sleep EEG-based brain age index based on Sun et al.[14]

EEG preprocessing: Participants underwent unattended in-home overnight PSG using the Compumedics System (Abbotsford, Victoria, Australia), with different models (SHHS: Series P; MrOS: Safiro; SOF: Siesta; MESA: Somte). We used two central EEG channels (C3-M2, C4-M1) for cohorts other than MESA and one central EEG channel (C4-M1) for MESA. EEG signals were notch-filtered at 60Hz, bandpass filtered at 0.5Hz to 35Hz, and then resampled to 200Hz. To minimize artifacts, we excluded 30-second epochs with maximum absolute amplitude >500uV or with more than 2 seconds of flat signal (SD <0.2uV).

Training Dataset: The data source was from Massachusetts General Hospital (MGH). Eligible participants were 18–80 years old, underwent full diagnostic PSG, and had a clinical diagnosis documented within five years before to one year after the PSG. EEGs with missing sleep staging were excluded. In total, 2,367 participants without significant neurological or psychiatric diseases (see supplementary Table 1 in that paper) were included as the training set. Sleep apnea patients were not excluded.

Feature Extraction: From each 30-second epoch, 102 time- and frequency-domain features were extracted as previously described (Sun et al., 2017). Features were averaged within each sleep stage, yielding 510 features per

© 2026 Sun H et al. *JAMA Network Open*.

participant. They were log-transformed and z-normalized using the training set parameters, and the same transformation was applied to the test set.

Feature Selection: In this alternative version implemented in Luna, we selected 13 EEG microstructure features from the original 510 features to reduce the noise. The selection was based on a subset of MGH participants with multiple diagnostic sleep recordings, from which we computed longitudinal precision and age correlations for each feature. Using stepwise feature selection, we identified a combination that achieves an optimal balance between longitudinal precision and age correlation. There was no subjective decision or bias involved.

Brain Age Model Training: The model minimizes an objective function J(w, b) with two terms: (1) mean squared prediction error; and (2) magnitude of the covariance between chronological age and BAI (brain age minus chronological age). There was strict separation of training and testing participants.

Interpretation: Variation in the correlation between brain age and chronological age across cohorts is expected and reflects differences in sample size, age distribution, and underlying health status. Notably, a very high correlation would imply that brain age simply replicates chronological age, rendering BAI uninformative. Instead, modest correlations reflect true deviations from normative neurophysiological aging—precisely the variation that BAI is designed to capture. These cohort-level differences do not stem from the model training procedure, which is constant across datasets.

Biological evidence of the sleep EEG features of BAI: Delta power, negatively associated with brain age[15], is often linked to sleep quality[16], synaptic homeostasis[17], and glymphatic clearance[18], all relevant to cognitive health. Spindle density, which is known to decline with age, reflects the integrity of thalamocortical circuits[19]. A reduction in spindle density in aging brains is associated with disrupted hippocampus-cortex communication, which is necessary for transferring memory into long-term storage[20]. Moreover, the loss of sleep spindles and spindle-SO coupling, both of which are linked to memory processing[21], have been associated with cognitive impairment and Alzheimer's disease (AD)[22], as captured by the BAI. Meanwhile, multiple kurtosis-based EEG features showed significant negative associations with incident dementia, including theta kurtosis in N3, sigma/delta/theta/alpha kurtosis in N2, and waveform kurtosis in N2. Kurtosis is a measure of heavy-tailness, i.e., the extent of large outliers. One possible hypothesis is that they are related to cyclic alternating pattern (CAP), where the A1 phase consists of K-complexes and slow waves (in delta band) followed by spindles (in sigma band), the A3 phase consists of arousals (in alpha band), and the A2 phase is an intermediate phase between A1 and A3. Overall, the temporal organization of CAP and non-CAP may contribute to the kurtosis features underlying BAI, although this requires future verification. In addition, the theta kurtosis in N2 and N3 could result from theta bursts (TBs) preceding slow oscillations[23], which have been associated with cognitive impairment and AD pathologies, including the cerebrospinal fluid amyloid β 42 to 40 ratio and tau levels[24]. Meanwhile, alpha power during N1, a transitional state between wakefulness and sleep, is positively associated with incident dementia and brain age[14]. This increase in alpha activity reflects lighter sleep depth at this transitional state. On the other hand, there are EEG microstructures that are associated with dementia, but are not selected into the BAI model, which may be due to insufficient age dependence. For example, slow oscillation spatial involvement is associated with prodromal AD[25]. Many REM patterns are associated with cognitive impairment and/or AD pathology, such as REM latency[26], theta power[27], and theta-delta slowing[28].

eReferences

1. Andersson C, Johnson AD, Benjamin EJ, Levy D, Vasan RS. 70-year legacy of the Framingham Heart Study. *Nature Reviews Cardiology*. 2019;16(11):687-698.

2. Quan SF, Howard BV, Iber C, et al. The sleep heart health study: design, rationale, and methods. *Sleep*. 1997;20(12):1077-1085.

3. Bild DE, Bluemke DA, Burke GL, et al. Multi-ethnic study of atherosclerosis: objectives and design. *American journal of epidemiology*. 2002;156(9):871-881.

4. Chen X, Wang R, Zee P, et al. Racial/ethnic differences in sleep disturbances: the Multi-Ethnic Study of Atherosclerosis (MESA). *Sleep*. 2015;38(6):877-888.

5. Fujiyoshi A, Jacobs Jr DR, Alonso A, Luchsinger JA, Rapp SR, Duprez DA. Validity of death certificate and hospital discharge ICD codes for dementia diagnosis: the Multi-Ethnic Study of Atherosclerosis. *Alzheimer Disease & Associated Disorders*. 2017;31(2):168-172.

© 2026 Sun H et al. *JAMA Network Open*.

6. Teng EL, Hasegawa K, Homma A, et al. The Cognitive Abilities Screening Instrument (CASI): a practical test for cross-cultural epidemiological studies of dementia. *International psychogeriatrics*. 1994;6(1):45-58.
7. Investigators A. The atherosclerosis risk in communit (ARIC) study: design and objectives. *American journal of epidemiology*. 1989;129(4):687-702.
8. Atherosclerosis Risk in Communities Study. Atherosclerosis risk in communities manual 20: surveillance of dementia in the ARIC cohort. Published online January 9, 2025.
9. Au R, Seshadri S, Wolf PA, et al. New norms for a new generation: cognitive performance in the framingham offspring cohort. *Experimental aging research*. 2004;30(4):333-358.
10. Satizabal CL, Beiser AS, Chouraki V, Chêne G, Dufouil C, Seshadri S. Incidence of dementia over three decades in the Framingham Heart Study. *New England journal of medicine*. 2016;374(6):523-532.
11. Blackwell T, Yaffe K, Ancoli-Israel S, et al. Associations between sleep architecture and sleep-disordered breathing and cognition in older community-dwelling men: the osteoporotic fractures in men sleep study. *Journal of the American Geriatrics Society*. 2011;59(12):2217-2225.
12. Teng E, Chui H. The modified mini-mental state examination (3MS). *Can J Psychiatry*. 1987;41(2):114-121.
13. Spira AP, Blackwell T, Stone KL, et al. Sleep-disordered breathing and cognition in older women. *Journal of the American Geriatrics Society*. 2008;56(1):45-50.
14. Sun H, Paixao L, Oliva JT, et al. Brain age from the electroencephalogram of sleep. *Neurobiology of aging*. 2019;74:112-120.
15. Sun H, Ye E, Paixao L, et al. The sleep and wake electroencephalogram over the lifespan. *Neurobiology of Aging*. 2023;124:60-70.
16. Long S, Ding R, Wang J, Yu Y, Lu J, Yao D. Sleep quality and electroencephalogram delta power. *Frontiers in Neuroscience*. 2021;15:803507.
17. Tononi G, Cirelli C. Sleep and synaptic homeostasis: a hypothesis. *Brain research bulletin*. 2003;62(2):143-150.
18. Fultz NE, Bonmassar G, Setsompop K, et al. Coupled electrophysiological, hemodynamic, and cerebrospinal fluid oscillations in human sleep. *Science*. 2019;366(6465):628-631.
19. Fernandez LM, Lüthi A. Sleep spindles: mechanisms and functions. *Physiological reviews*. 2020;100(2):.
20. Klinzing JG, Niethard N, Born J. Mechanisms of systems memory consolidation during sleep. *Nature neuroscience*. 2019;22(10):1598-1610.
21. Muehlroth BE, Sander MC, Fandakova Y, et al. Precise slow oscillation–spindle coupling promotes memory consolidation in younger and older adults. *Scientific reports*. 2019;9(1):1940.
22. Páez A, Gillman SO, Dogaheh SB, et al. Sleep spindles and slow oscillations predict cognition and biomarkers of neurodegeneration in mild to moderate Alzheimer’s disease. *Alzheimer’s & Dementia*. 2025;21(2):e14424.
23. Gonzalez CE, Mak-McCully RA, Rosen BQ, et al. Theta bursts precede, and spindles follow, cortical and thalamic downstates in human NREM sleep. *Journal of Neuroscience*. 2018;38(46):9989-10001.
24. Pulver RL, Kronberg E, Medenblik LM, et al. Mapping sleep’s oscillatory events as a biomarker of Alzheimer’s disease. *Alzheimer’s & Dementia*. 2024;20(1):301-315.
25. Sharon O, Zhelezniakov V, Gat Y, et al. Slow wave synchrony during NREM sleep tracks cognitive impairment in prodromal Alzheimer’s disease. *Alzheimer’s & Dementia*. 2025;21(5):e70247.
26. Jin J, Chen J, Cavaillès C, et al. Association of rapid eye movement sleep latency with multimodal biomarkers of Alzheimer’s disease. *Alzheimer’s & Dementia*. 2025;21(2):e14495.
27. André C, Champetier P, Rehel S, et al. Rapid eye movement sleep, neurodegeneration, and amyloid deposition in aging. *Annals of neurology*. 2023;93(5):979-990.
28. Lam AKF, Carrick J, Kao CH, et al. Electroencephalographic slowing during REM sleep in older adults with subjective cognitive impairment and mild cognitive impairment. *Sleep*. 2024;47(6).

eTable 1. Covariates Used in Each Cohort

Name	MESA	ARIC	FHS-OS	MrOS	SOF
Age at sleep study	Yes	Yes	Yes	Yes	Yes
Sex	Yes	Yes	Yes	Not used since all males	Not used since all females
Race/Ethnicity	Asian (Chinese), Black, Hispanic, White	Not used since all White	White, Non-White	White, Non-White	Not used since only 1 participant is non-White
Education higher than college	From Exam 1	From SHHS sleep study		From Visit 1	From Visit 1
Body mass index at sleep study	From Exam 5, closest to sleep study	From SHHS sleep study		From Sleep Visit 1	From Visit 8, closest to sleep study
Current smoking	From Exam 5, closest to sleep study	From SHHS sleep study		From Sleep Visit 1	From Visit 8, closest to sleep study
Sleep medication	Pittsburgh Sleep Quality Index: take sleeping pill ≥ 1/week, from pre-sleep questionnaires	benzodiazepine, tricyclic anti-depressants (TCA), or non-TCA (other than monoamine oxidase inhibitor), within two weeks of the SHHS sleep study		Pittsburgh Sleep Quality Index: take sleeping pill ≥ 1/week, from Sleep Visit 1	Reported taking any medication for sleep (OTC, Rx) in the past 30 days, from Visit 8, closest to sleep study
Physical activity level	Total walking time (minutes / week) from Exam 5, closest to sleep study	Self-reported sport index from Visit 3	Physical Activity Score (PAS) from Exam 7	Physical Activity Score (PAS) from Sleep Visit 1	Walk one or more block without stopping for exercise, from Visit 8, closest to sleep study
Comorbidity	Diabetes, hypertension, and depression from Exam 5, closest to sleep study; heart attack and stroke from surveillance data before sleep study	From the SHHS sleep study		from Sleep Visit 1	from Visit 8, closest to sleep study

© 2026 Sun H et al. *JAMA Network Open*.

eTable 2. Cumulative Number of Participants Who Developed Dementia or Died Over 25 Years

Time since sleep study (Year)	MESA (#dementia, #death)	ARIC (#dementia, #death)	FHS-OS (#dementia, #death)	MrOS (#dementia, #death)	SOF (#dementia, #death)	Total (#dementia, #death)
1	4, 17	0, 9	0, 1	39, 0	0, 0	43, 27
2	9, 30	1, 18	1, 1	156, 21	0, 0	167, 70
3	17, 50	2, 32	2, 2	177, 75	0, 0	198, 159
4	25, 68	3, 57	3, 7	273, 141	12, 0	316, 273
5	67, 89	3, 68	4, 12	300, 207	57, 2	431, 378
6	103, 112	4, 87	8, 13	320, 287	86, 4	521, 503
7	117, 136	7, 102	10, 21	350, 356	86, 10	570, 625
8	119, 138	9, 126	13, 26	370, 438	86, 12	597, 740
9	119, 138	17, 146	16, 34	370, 549	86, 12	608, 879
10	119, 138	25, 177	18, 44	373, 650	86, 12	621, 1021
11	119, 138	35, 204	20, 51	429, 747	86, 12	689, 1152
12	119, 138	46, 227	24, 60	469, 806	86, 12	744, 1243
13	119, 138	54, 258	27, 66	470, 813	86, 12	756, 1287
14	119, 138	70, 290	33, 71	470, 813	86, 12	778, 1324
15	119, 138	94, 328	37, 83	470, 813	86, 12	806, 1374
16	119, 138	142, 361	43, 89	470, 813	86, 12	860, 1413
17	119, 138	177, 392	49, 100	470, 813	86, 12	901, 1455
18	119, 138	197, 423	51, 113	470, 813	86, 12	923, 1499
19	119, 138	230, 448	57, 126	470, 813	86, 12	962, 1537
20	119, 138	274, 472	58, 132	470, 813	86, 12	1007, 1567
21	119, 138	311, 501	59, 141	470, 813	86, 12	1045, 1605
22	119, 138	339, 539	59, 150	470, 813	86, 12	1073, 1652
23	119, 138	353, 561	59, 155	470, 813	86, 12	1087, 1679
24	119, 138	354, 565	59, 158	470, 813	86, 12	1088, 1686
25	119, 138	354, 565	59, 158	470, 813	86, 12	1088, 1686

© 2026 Sun H et al. *JAMA Network Open*.

eTable 3. Participant Characteristics for Low Brain Age Index (BAI) (≤ –3 Years) vs High BAI (≥3 Years)

In unadjusted analyses, participants with a lower BAI tended to be older across all cohorts (except SOF due to small sample size or the limited age range). No sex difference was observed. BMI was higher in the higher BAI group in MESA (28.2 vs. 29.1 kg/m², p=0.02) and MrOS (26.8 vs. 28.2 kg/m2, p<0.001). AHI was consistently lower in the higher BAI group in MESA, ARIC, and MrOS.

Variable (definitions follow eTable 1)	Low BAI (≤ -3y)	High BAI (≥ +3y)
**MESA**		
N	1010	229
Age at sleep study, years	72.3 (9.4)	62.9 (5.7)
Sex (Female)	538 (53.3%)	109 (47.6%)
BMI, kg/m^2	28.2 (5.3)	29.1 (6.1)
Current smoker	53 (5.2%)	27 (11.8%)
College degree	382 (37.9%)	92 (40.2%)
APOE e4 carrier	253 (26.4%)	61 (27.9%)
AHI, /hour	15.1 (16.9)	11.8 (13.0)
Sleep medication use	106 (10.6%)	34 (15.1%)
Hypertension	594 (58.9%)	123 (53.7%)
Diabetes	209 (20.9%)	48 (21.1%)
Myocardial infarction	56 (5.5%)	7 (3.1%)
Stroke	16 (1.6%)	3 (1.3%)
Depression	137 (13.9%)	39 (17.3%)
**ARIC**		
N	546	572
Age at sleep study, years	62.8 (5.9)	62.1 (5.4)
Sex (Female)	288 (52.7%)	286 (50.0%)
BMI, kg/m^2	28.7 (5.2)	28.7 (5.0)
Current smoker	44 (8.1%)	62 (10.8%)
College degree	250 (45.8%)	248 (43.4%)
AHI, /hour	11.0 (14.7)	8.6 (10.9)
Sleep medication use	48 (8.8%)	72 (12.6%)
Hypertension	180 (33.0%)	234 (40.9%)
Diabetes	26 (4.8%)	37 (6.5%)
Myocardial infarction	39 (7.2%)	38 (6.7%)
Stroke	11 (2.0%)	19 (3.3%)
Depression	7 (1.5%)	23 (4.5%)
**FHS-OS**		
N	175	246
Age at sleep study, years	60.3 (9.0)	57.3 (8.5)
Sex (Female)	85 (48.6%)	117 (47.6%)
BMI, kg/m^2	28.5 (4.9)	28.8 (5.5)

© 2026 Sun H et al. *JAMA Network Open*.

Current smoker	18 (10.3%)	39 (15.9%)
College degree	106 (60.6%)	160 (65.0%)
APOE e4 carrier	26 (23.0%)	23 (17.0%)
AHI, /hour	10.1 (15.7)	8.7 (11.5)
Sleep medication use	8 (4.6%)	31 (12.6%)
Hypertension	57 (32.6%)	71 (28.9%)
Diabetes	8 (4.7%)	15 (6.1%)
Myocardial infarction	9 (5.2%)	11 (4.5%)
Stroke	2 (1.1%)	3 (1.2%)
Depression	3 (2.0%)	13 (6.1%)
**MrOS**		
N	1168	429
Age at sleep study, years	77.6 (5.6)	73.5 (4.2)
BMI, kg/m^2	26.8 (3.6)	28.2 (4.2)
Current smoker	22 (1.9%)	14 (3.3%)
College degree	926 (79.3%)	326 (76.0%)
APOE e4 carrier	241 (23.0%)	100 (26.8%)
AHI, /hour	13.7 (13.8)	11.3 (12.5)
Sleep medication use	159 (13.6%)	71 (16.6%)
Hypertension	564 (48.3%)	228 (53.1%)
Diabetes	143 (12.2%)	74 (17.2%)
Myocardial infarction	199 (17.0%)	67 (15.6%)
Stroke	43 (3.7%)	15 (3.5%)
Depression	64 (5.5%)	35 (8.2%)
**SOF**		
N	166	11
Age at sleep study, years	82.9 (3.0)	81.5 (2.8)
BMI, kg/m^2	27.2 (4.3)	29.3 (5.2)
Current smoker	3 (1.8%)	0 (0%)
College degree	131 (78.9%)	9 (81.8%)
APOE e4 carrier*	8 (8.5%)	1 (12.5%)
AHI, /hour	14.3 (14.6)	11.7 (8.8)
Sleep medication use	29 (17.5%)	1 (9.1%)
Hypertension	95 (57.2%)	6 (54.5%)
Diabetes	14 (8.4%)	2 (18.2%)
Myocardial infarction	24 (14.5%)	1 (9.1%)
Stroke	20 (12.0%)	3 (27.3%)
Depression	16 (9.6%)	1 (9.1%)

* the participants with APOE genotyping in SOF came from only one study site. BMI – body mass index. APOE – apolipoprotein E. BAI – Sleep EEG-based brain age index. AHI – Apnea-hypopnea index.

© 2026 Sun H et al. *JAMA Network Open*.

eFigure 1. Illustration of Example Sleep Electroencephalographic Microstructure Features

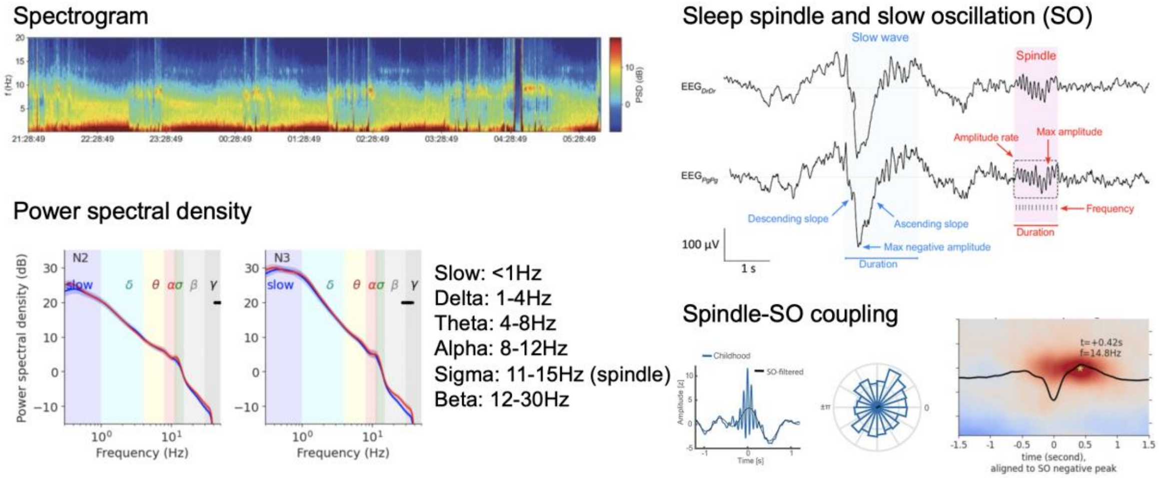

© 2026 Sun H et al. *JAMA Network Open*.

eFigure 2. Forest Plots of Associations Between Brain Age Index and Incident Dementia While Adjusting for Many (Overcomplete) Covariates

Study	HR	95% CI	P	Hazard Ratio	Weight
MESA	1.222	[0.843; 1.772]	0.289		13.4%
ARIC	1.293	[0.995; 1.680]	0.054		27.0%
FHS-OS	2.318	[1.249; 4.300]	0.008		4.9%
MrOS	1.219	[1.003; 1.481]	0.047		48.8%
SOF	1.855	[1.058; 3.252]	0.031		5.9%
**Random effects model**	**1.310**	**[1.143; 1.501]**	**< 0.001**		**100.0%**

Heterogeneity: $I^2 = 26\%$, $\tau^2 < 0.0001$, $p = 0.25$

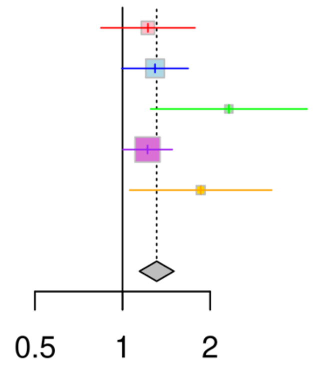

Forest plots of the associations between brain age index and incident dementia while adjusting for many (overcomplete) covariates, including age, sex, baseline cognitive score, education level, body mass index (BMI), current smoking status, race, sleep medication, physical exercise level, diabetes, hypertension, heart attack, stroke, depression, and the apnea-hypopnea index, as a sensitivity analysis for the association between brain age index (BAI) and incident dementia. HR – hazards ratio. MESA – Multi-Ethnic Study of Atherosclerosis. ARIC – Atherosclerosis Risk in Communities. FHS-OS – Framingham Heart Study Offspring. MrOS – Osteoporotic Fractures in Men. SOF – Study of Osteoporotic Fractures.

© 2026 Sun H et al. *JAMA Network Open*.

eFigure 3. Forest Plots of Associations Between Brain Age Index and Dementia While Adjusting and Not Adjusting for *APOE* ε4 Allele Carrier Status

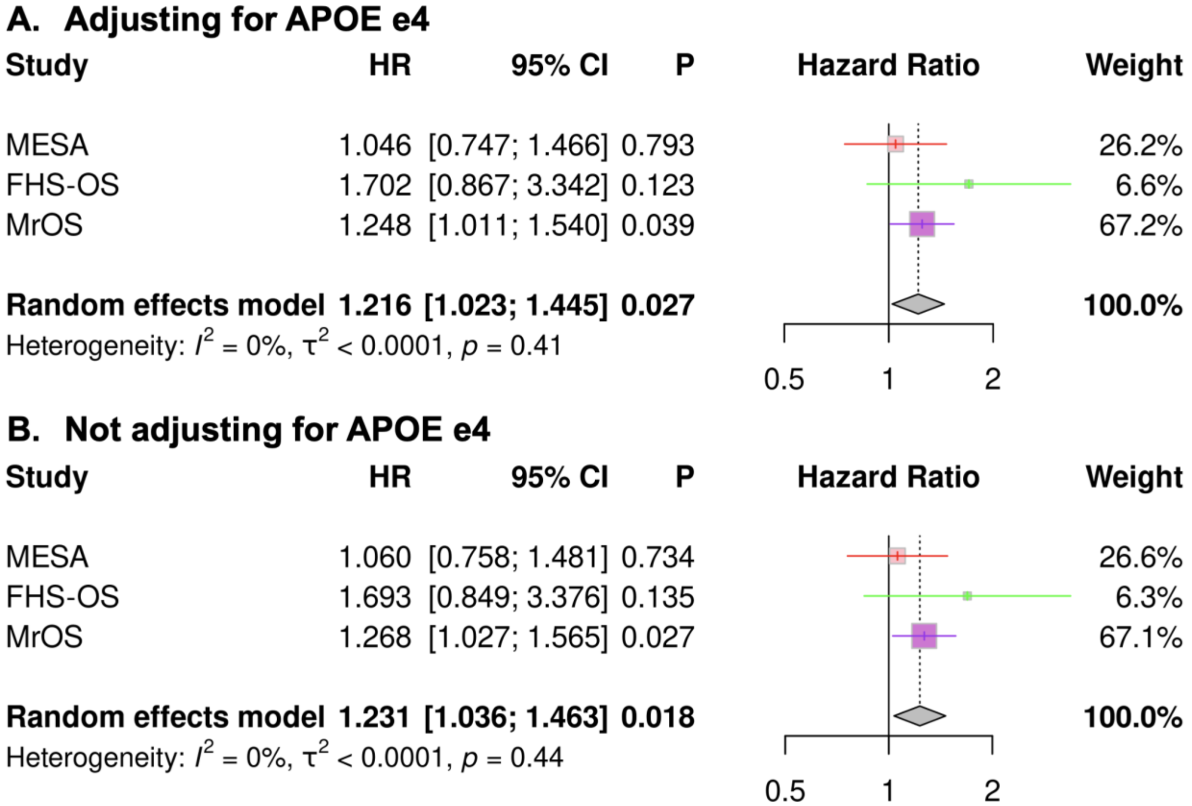

For this analysis, the SOF cohort was excluded since the participants with APOE genotyping in SOF came from only one study site. Both analyses, with and without APOE, were performed based on the same subset of participants with APOE genotypes available. CI – confidence interval. HR – hazards ratio. MESA – Multi-Ethnic Study of Atherosclerosis. ARIC – Atherosclerosis Risk in Communities. FHS-OS – Framingham Heart Study Offspring. MrOS – Osteoporotic Fractures in Men. SOF – Study of Osteoporotic Fractures. * Only including the three cohorts with complete data on APOE e4 status.

© 2026 Sun H et al. *JAMA Network Open*.

eFigure 4. Forest Plots of Associations Between Brain Age Index and Incident Dementia Stratified by Sex and Age

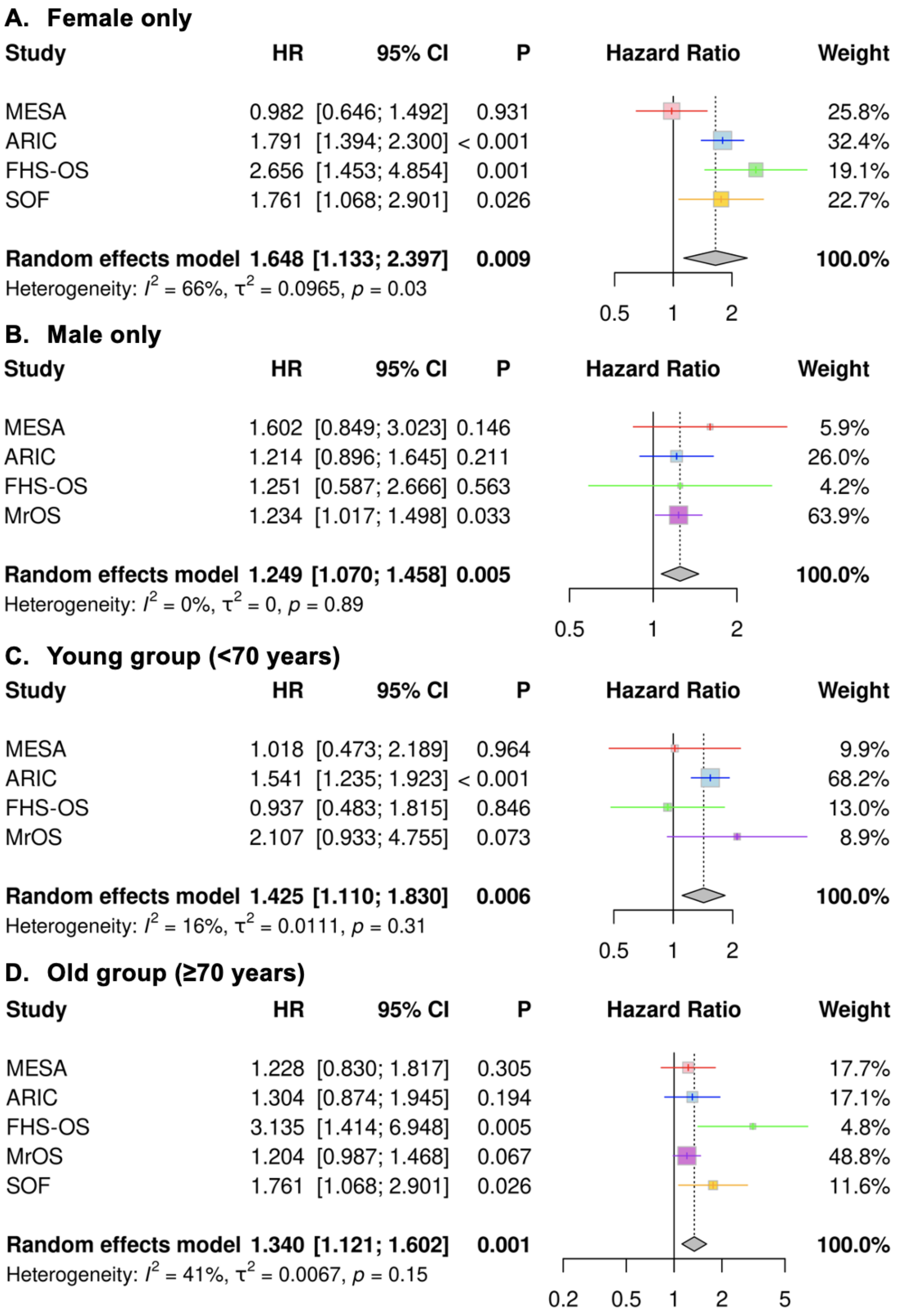

Forest plots of the associations between brain age index and incident dementia while stratified by sex (a-b) and age (c-d). Results were statistically significant across sex and age groups. SOF has all females. MrOS has all males. SOF has no participants under

© 2026 Sun H et al. *JAMA Network Open*.

70 years old. CI – confidence interval. HR – hazards ratio. MESA – Multi-Ethnic Study of Atherosclerosis. ARIC – Atherosclerosis Risk in Communities. FHS-OS – Framingham Heart Study Offspring. MrOS – Osteoporotic Fractures in Men. SOF – Study of Osteoporotic Fractures.

© 2026 Sun H et al. *JAMA Network Open*.

eFigure 5. Forest Plots of the Associations Between Brain Age Index and Dementia While Excluding the MESA Cohort

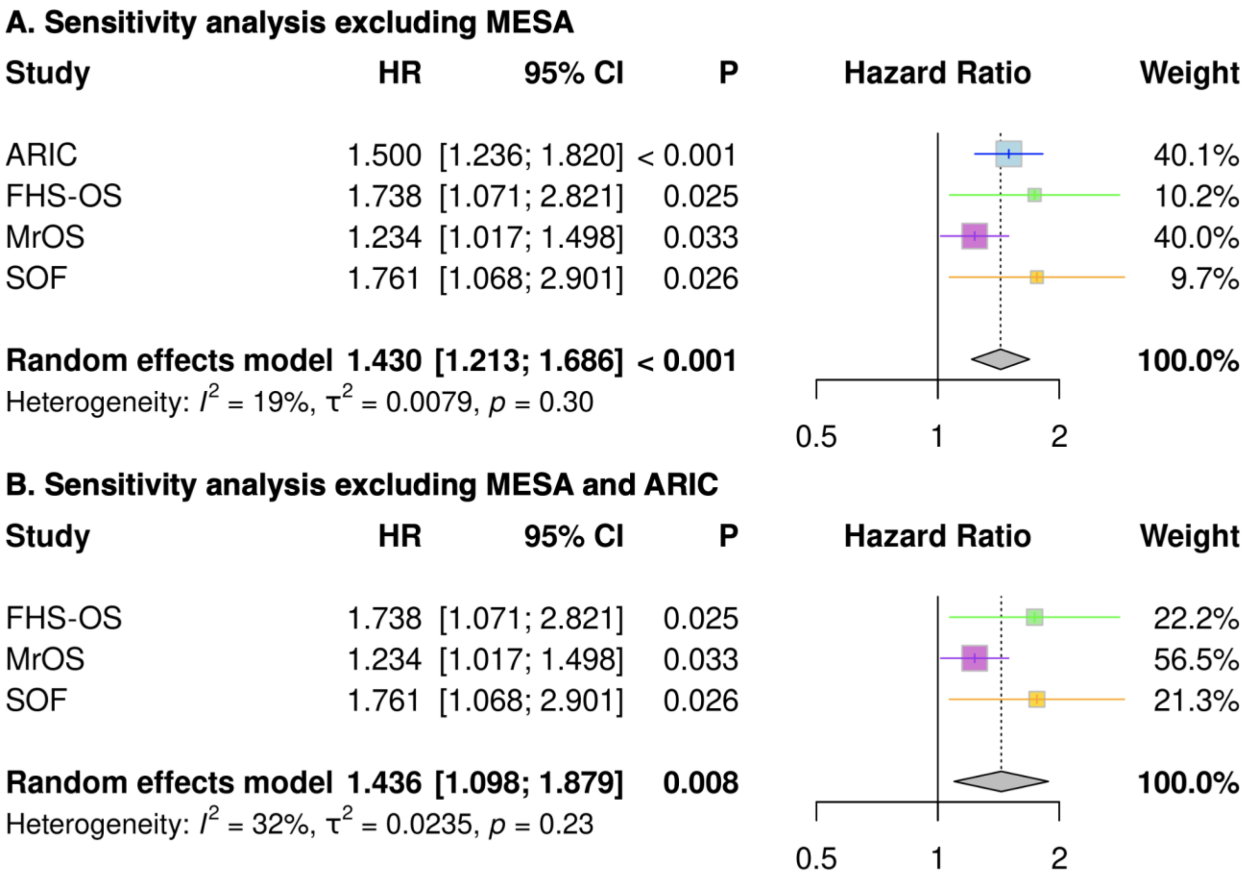

Forest plots of the associations between brain age index and dementia while (a) excluding MESA since the dementia outcome was ascertained involving ICD codes during hospitalization; (b) excluding both MESA and ARIC since deaths were primarily ascertained from hospital records. CI – confidence interval. HR – hazards ratio. MESA – Multi-Ethnic Study of Atherosclerosis. ARIC – Atherosclerosis Risk in Communities. FHS-OS – Framingham Heart Study Offspring. MrOS – Osteoporotic Fractures in Men. SOF – Study of Osteoporotic Fractures.

© 2026 Sun H et al. *JAMA Network Open*.
