# Supplementary material for: Machine Learning–Based Sleep Electroencephalographic Brain Age Index and Dementia Risk: An Individual Participant Data Meta-Analysis
Source: JAMA Netw Open. 2026 Mar 19;9(3):e261521. doi: 10.1001/jamanetworkopen.2026.1521 (PMC13003368; doi:10.1001/jamanetworkopen.2026.1521)
Supplement: Supplement 2. — Data Sharing Statement [file jamanetwopen-e261521-s002.pdf]

Data Sharing Statement

Sun. Machine Learning–Based Sleep Electroencephalographic Brain Age Index and Dementia Risk. *JAMA Netw Open*. Published March 19, 2026. doi:10.1001/jamanetworkopen.2026.1521

Data
Data available: No

Additional Information
Explanation for why data not available: This is a secondary analysis of existing data. Data can be requested and accessed through the established data request process from each original study.
